# New Irish Midwifery Registration Standards and the Missed Opportunity for Gender‐Inclusive Curricula and Reproductive Justice

**DOI:** 10.1111/birt.70097

**Published:** 2026-07-18

**Authors:** John P. Gilmore, Anita Byrne, Noah Halpin, Ciara Kirwan, Elizabeth Newnham, Sally Pezaro, Amy Rochford, Ky Walker, Jeannine Webster

**Affiliations:** ^1^ School of Nursing, Midwifery and Health Systems University College Dublin Dublin Ireland; ^2^ Dundalk Institute of Technology Dundalk Co. Louth Ireland; ^3^ Transgender Equality Network Ireland Dublin Ireland; ^4^ College of Nursing and Health Sciences; Caring Futures Institute Flinders University Adelaide Australia; ^5^ Research Centre for Healthcare and Communities Coventry University Coventry UK; ^6^ Rotunda Hospital Dublin Ireland; ^7^ Trans Healthcare Action Dublin Ireland; ^8^ School of Public Health University College Cork Cork Ireland

**Keywords:** curriculum reform, gender inclusion, human rights, midwifery education, trans and gender diverse health, transgender

## Abstract

The 2025 revision of Ireland's midwifery education standards marks the first comprehensive update since 2012 and includes an important step forward in acknowledging trans and gender diverse (TGD) people as recipients of midwifery care. However, despite this progress, the standards remain constrained by gender‐exclusive language and a lack of substantive integration of TGD perspectives across curricula. Drawing on international literature and recent evidence on TGD pregnancy experiences, this commentary argues that recognition without structural embedding risks tokenism and perpetuates inequity. Situating the discussion within a human rights framework, it highlights the relevance of the International Confederation of Midwives' Philosophy and Bill of Rights, which centers on self‐determination, bodily autonomy, justice and equity, principles that align directly with trans inclusion. The commentary calls for consistent inclusive terminology, explicit competencies in TGD health, and leadership education that equips midwives to advocate for equity and human rights. It also underscores the need to foster safe, affirming environments for trans and non‐binary midwives, ensuring that the profession itself embodies the inclusivity it promotes in care.

## 1 Introduction

High‐quality curricula are the foundation of safe, effective, and equitable midwifery practice. The content and framing of midwifery education shape not only the knowledge and skills of graduates but also the values, language, and assumptions they carry into clinical practice. Internationally, questions of how curricula prepare midwives to meet the needs of increasingly diverse populations have become urgent, particularly in relation to trans and gender diverse (TGD) people who continue to experience significant barriers in accessing respectful perinatal care [1]. Midwives want to provide equitable and just care, but lack a roadmap to do so. Ensuring that midwifery education reflects inclusivity is therefore not an optional addition but a core requirement of compassionate, person‐centered practice.

In this context, the launch of revised midwifery education standards in Ireland in 2025, the first comprehensive update since 2012 [2], is of note not only in Ireland but also internationally. This long‐awaited review aims to address longstanding concerns about gaps in practice preparation, including distinguishing midwifery as a distinct profession, embracing holistic care, and providing the continuum of midwifery care beyond the hospital environment [3, 4]. As such, these standards have a unique opportunity to be trailblazing in advancing the profession and meeting the evolving needs of those who give birth through 2025 and beyond.

## 2 Linguistic Erasure and Its Consequences

Importantly, the new standards explicitly acknowledge TGD people as recipients of midwifery care and highlight the need for compassionate attention to their specific needs. This recognition is a welcome and important step, signaling a shift in awareness that TGD people are legitimate recipients of midwifery care whose experiences must be reflected within the education of future midwives in line with standards from the International Confederation of Midwives [5]. However, this welcome development is undermined by the persistence of gender‐exclusive terminology throughout the standards. The document consistently employs the terms “women” and “mothers” without further qualification or inclusivity. This creates a fundamental dissonance: while TGD people are acknowledged in principle, the structural framing of the standards excludes them. Such inconsistency risks tokenism, where reference to TGD populations exists only symbolically rather than being embedded across educational and professional expectations.

The consequences of this inconsistency are significant. Trans masculine and non‐binary people who give birth are not women, and their identities and health needs cannot be collapsed into “female”‐centered narratives of perinatal care. Exclusively gendered terminology perpetuates marginalization, erases TGD people from curricula and resources, and risks leaving graduates underprepared for inclusive practice.

While the erasure of trans masculine and non‐binary people in perinatal contexts is well recognized, trans feminine people are also frequently excluded from midwifery and reproductive health discourse. Although midwives in Ireland primarily focus on pregnancy and childbirth, their scope also encompasses sexual health promotion, contraception, fertility awareness, health counseling, and wider reproductive wellbeing, all of which are relevant to trans feminine people. Trans feminine individuals may seek fertility preservation counseling, contraceptive advice, or sexual and reproductive health education, yet they are rarely acknowledged within midwifery curricula or practice frameworks. Inclusive midwifery education should therefore not only address those who give birth, but also encompass the diversity of people engaging with midwifery and reproductive health services, ensuring that trans feminine people are recognized and supported within the continuum of care.

Gender‐inclusive language is not a matter of semantics but of ensuring safety, equity, and dignity [6]. Inclusive terminology supports trust, reduces barriers to care, and strengthens the midwifery profession's capacity to deliver person‐centered care.

## 3 Structural Cisnormativity in Midwifery Education

Evidence from a recent systematic scoping review of the pregnancy experiences of TGD people [7] underscores why curricular change is so urgent. The review found that while pregnancy experiences are complex and heterogeneous, they are consistently shaped by cisnormative assumptions that frame pregnancy as an exclusively cisgender female experience. Such assumptions not only erase the reality of TGD pregnancies but also position them as illegitimate or even impossible within clinical and educational contexts. In practice, this erasure manifests in administrative and clinical barriers, such as difficulties in registering a pregnant man, as well as in the silencing or delegitimization of TGD pregnancy narratives within health professions education.

Importantly, the review highlights that institutional cisnormativity and trans‐negativity are not incidental but structural features of maternal and newborn care. Dominant ideologies link genotypic sex to phenotypical realities and socially constructed categories of gender, producing exclusion in consultation rooms, in curricula, and in the broader organization of maternity care. This not only marginalizes TGD people but also embeds bias within the systems that govern care delivery and education. The consequences extend beyond erasure: such frameworks can cultivate unsafe and unjust care environments that reproduce reproductive injustices and can amount to obstetric violence [6].

The review findings reinforce the argument that midwifery education must move beyond superficial inclusion. While isolated training initiatives or elective modules may provide individual midwives with knowledge and skills, they are insufficient to dismantle the cisnormative underpinnings of current curricula. What is needed instead are structural and transformative approaches that reexamine how “sex,” “gender,” and “pregnancy” are conceptualized in midwifery knowledge, and that apply intersectional and critical diversity frameworks to understand how TGD pregnancy experiences intersect with other forms of marginalization, including racism, classism, and ageism [7]. Without such structural reform, curricular updates risk reproducing inequities rather than addressing them.

## 4 Challenging Ideological Constraints: The Problem of “Sexed Language”

We are further concerned about the invocation of “sexed language” as a supposed safety consideration, most prominently advanced by Gribble et al. [8]. Such arguments are not supported by empirical evidence and, in practice, undermine safety by erasing those whose identities do not align with cisnormative assumptions; the belief, often implicit, that all people identify with the sex they were assigned at birth [9].

Safe and effective midwifery education is best served by inclusivity and compassion, not restrictive discourses that prioritize ideology over evidence. It is therefore critical to address now, as these registration standards will shape the education of the next generation of midwives. The standards clearly identify the requirement for midwives to uphold the highest ethical standards, “ensuring that every aspect of care is grounded in dignity, respect, privacy, confidentiality, equity, and justice” – to do this all must be included [2].

## 5 Rights‐Based Midwifery Education

The revised standards must also be considered within the broader framework of healthcare rights for sexual and gender minorities. The right to healthcare is inseparable from other human, civil, and social rights and cannot be realized unless services are both appropriate and culturally responsive [10]. In the context of midwifery, this means that naming TGD people within educational standards is insufficient if the curriculum itself does not prepare graduates to deliver affirming, equitable, and inclusive care. Without embedding the principles of equality and nondiscrimination, universal healthcare, reproductive rights, emancipation, and justice cannot be achieved in practice.

The International Confederation of Midwives Philosophy and Model of Midwifery Care explicitly state that midwifery recognizes “the right to self‐determination, and is respectful, personalized, continuous and non‐authoritarian,” and that it is “based on the ethical principles of justice, equity, and respect for human dignity” [11]. These principles are not only compatible with trans inclusion but essential to it. Language from the ICM framework can be powerfully used to speak to trans liberation, particularly in dialog with midwifery leaders, by connecting the profession's commitment to bodily autonomy and personalized care to the rights and experiences of trans people. The ICM Bill of Rights for Women and Midwives also affirms the right to bodily autonomy, freedom from discrimination, and informed consent, all values that are inseparable from trans justice and liberation [12].

In advancing trans inclusion within midwifery education, it is also vital to recognize that trans and non‐binary midwives exist and are part of the profession. Too often, discussions of inclusion focus solely on trans and gender diverse people as recipients of care, overlooking the experiences of those who work within midwifery. Any genuine commitment to equity and self‐determination within midwifery must therefore extend to creating safe, affirming environments for trans and non‐binary students, educators, and practitioners, ensuring that the profession itself embodies the inclusivity it seeks to deliver in care.

Midwives cannot be taught to uphold autonomy and self‐determination for some groups while denying these same rights to others. These foundational principles of midwifery care demand inclusivity, and they position midwives as ideally placed advocates for trans‐affirming, rights‐based, and person‐centered care that honors the dignity and authenticity of all people who give birth [13, 14].

## 6 From Recognition to Action: Recommendations for Curricular Reform

To move beyond partial recognition, we recommend the following:

- Adopting gender‐inclusive terminology consistently across the standards, such as “pregnant people” or “people who give birth,” exclusively or in parallel with the terms “women” and “mothers.”
- Embedding explicit competencies within the curriculum to prepare midwives to provide safe, inclusive, and affirming care for TGD people, including awareness of barriers to access, respectful communication, and culturally competent clinical practice.
- Integrating TGD perspectives throughout the document, rather than relegating them to a single reference, thereby reflecting the diversity of those who require midwifery care.
- Rejecting “sexed language” framings such as those advanced by Gribble et al. [8], which are not evidence‐based and risk entrenching exclusion, instead ensuring that safety and quality of care are grounded in inclusivity and compassion.

## 7 Leadership and the Future of Midwifery Education

A further focal point of the revised standards is the enhanced emphasis on leadership. This aligns with a growing and global body of literature that has begun to map the characteristics and enablers of strong midwifery leadership. Recent findings identify vision, compassion, authenticity, the cultivation of strategic relationships, and a strong professional identity as essential qualities, while enablers include professional development, succession planning, and interprofessional collaboration [15]. These insights echo wider frameworks on transformational and compassionate leadership in healthcare, which emphasize empathy, equity, and advocacy as central to creating positive cultures of practice [11].

Positioning leadership as a cornerstone of midwifery education is therefore welcome. However, we contend that the absence of fully embedded recognition of TGD people within the revised standards undermines this leadership agenda. Leadership in midwifery must not be confined to administrative or technical domains but must prepare midwives to lead inclusively, challenge inequities, and advocate for all service users. Without this, the leadership focus risks reproducing narrow and exclusionary understandings of who midwifery care is for. Embedding inclusivity into leadership preparation would instead strengthen the profession's capacity to deliver compassionate, equitable, and person‐centered care at national and international levels.

## 8 Conclusion

Ultimately, the revised Irish standards therefore represent both progress and missed opportunity. While they advance recognition of TGD people in name, they fail to challenge entrenched gender‐exclusive frameworks. Genuine progress requires both acknowledgement and integration: inclusive terminology, comprehensive competencies, and curricular design that reflects the realities of all people who give birth. As visibility and compassion for TGD communities grows, we urge regulators, educators, and practitioners to move beyond partial recognition and toward genuine inclusion and authentic allyship [13]. Future iterations of the standards must be more courageous in aligning midwifery education with principles of equity, human rights, justice and emancipation. We will continue to advocate strongly for a midwifery curriculum that reflects the realities of all people who give birth and ensures that their needs are not only acknowledged but fully met.

This discussion has clear international relevance. Midwives across diverse contexts are grappling with the question of how to ensure inclusivity and equity in perinatal care. The Irish example illustrates how regulatory recognition must be matched by structural change to avoid tokenism and to fulfill the profession's ethical obligation to provide safe, compassionate, and person‐centered care to all and the progressive nature of midwifery as it evolves alongside the populations it serves.

## Author Contributions

**John P. Gilmore:** conceptualization, writing – original draft, writing – review and editing. **Anita Byrne:** writing – review and editing. **Noah Halpin:** writing – review and editing. **Ciara Kirwan:** writing – review and editing. **Elizabeth Newnham:** writing – review and editing. **Sally Pezaro:** writing – review and editing. **Amy Rochford:** writing – review and editing. **Ky Walker:** writing – review and editing. **Jeannine Webster:** writing – review and editing.

## Funding

The authors have nothing to report.

## Ethics Statement

This article is a commentary and does not involve original research with human participants or the use of identifiable patient information. Consequently, ethical approval and informed consent were not required.

## Conflicts of Interest

A.B. sat on the advisory committee for the drafting of the Registered Midwife Programmes Standards. All other authors declare no conflicts of interest.

## Data Availability

Data sharing is not applicable to this article as no datasets were generated or analysed during the current study.
